# Assessment of cardiac and vessel functions in childhood psoriasis

**DOI:** 10.3906/sag-1812-139

**Published:** 2019-04-18

**Authors:** Berna ŞAYLAN ÇEVİK, Figen AKALIN, Elif EROLU, Seçkin GENÇOSMANOĞLU, Tülin ERGUN

**Affiliations:** 1 Department of Pediatric Cardiology, School of Medicine, Marmara University, İstanbul Turkey; 2 Department of Pediatric Cardiology, Ümraniye Research Hospital, İstanbul Turkey; 3 Department of Dermatology, School of Medicine, Marmara University, İstanbul Turkey

**Keywords:** Psoriasis, childhood, echocardiography, ventricular strain, arterial elasticity

## Abstract

**Background/aim:**

Psoriasis is a chronic inflammatory disease. The effect of psoriasis on the cardiovascular system has not been studied in children before. We studied ventricular strain and vascular functions to assess early cardiovascular effects of psoriasis during childhood.

**Materials and methods:**

The study population consisted of 20 psoriatic and 20 age- and sex-matched control subjects. Two-dimensional echocardiography images, longitudinal and global strain, and carotid and brachial ultrasound studies were performed.

**Results:**

The mean age of psoriatic children was 14 ± 0.89 years and that of the controls was 14.05 ± 0.88. There were significant increases in terms of interventricular septum diastolic and left ventricular posterior wall diastolic diameter and decreases in mitral E, mitral A, and E/A values between groups. Tissue Doppler imaging revealed significant differences between groups in terms of lateral annulus E’, A’, E’/A, isovolumetric contraction time, and ejection time. Aortic stiffness was significantly higher and global circumferential strain and longitudinal strain were significantly lower in the psoriasis group. Carotid intima media thickness and flow-mediated dilatation did not differ significantly between the groups.

**Conclusion:**

Cardiac left ventricular and arterial functions are affected in psoriatic children and may be an alarming sign of atherosclerotic heart disease in the long term. Early detection of these changes may be helpful for eliminating other risk factors.

## 1. Introduction

Psoriasis is one of the most common immune-mediated chronic inflammatory diseases among adults, affecting nearly 1%–4% of the population (1). There are increasing data about the effects of psoriasis on the cardiovascular system and development of atherosclerotic vascular changes. 

The role of inflammation in atherosclerosis pathogenesis has been studied extensively. With the chronic inflammatory status leading to the release of inflammatory and proatherogenic mediators, arterial functions are altered and atherosclerotic changes are promoted (2).

Previous studies conducted in adult psoriatic patients have shown impaired ventricular functions, decreased ventricular strain, increased carotid intima media thickness (CIMT), and decreased flow-mediated dilatation (FMD) of the brachial artery. Echocardiographic methods such as tissue Doppler imaging (TDI) and strain echocardiography are beneficial methods in detection of subtle changes in myocardial performance that cannot be identified by routine echocardiographic examination. Magnetic resonance imaging and scintigraphy were also used to detect left ventricular systolic function in adult psoriatic patients. These advanced techniques may be used in detection of early cardiac effects of psoriasis during childhood (3). On the other hand, ongoing inflammation in psoriatic patients may accelerate the atherosclerotic process, leading to impaired vascular functions such as aortic strain, distensibility, and stiffness, which results in compromised left ventricular function (4).

The aim of this study is to assess cardiac involvement in pediatric cases by evaluating subclinical myocardial changes and arterial functions using TDI, strain echocardiography, arterial distensibility, stiffness, CIMT, and FMD. 

## 2. Materials and methods

### 2.1. Study population

The study group was recruited from the patients under follow-up with a diagnosis of pediatric psoriasis in outpatient clinics of the Marmara University Dermatology Department. The control group consisted of healthy children. Informed consent was received from all patients and controls. The study was approved by the local ethics committee of the Marmara University School of Medicine (Date: 02.02.2018/09.2018.114).

Twenty consecutive children with a diagnosis of psoriasis vulgaris (12 girls, 8 boys, mean age: 14.2 ± 0.89) and 20 sex- and age-matched controls (12 girls, 8 boys, mean age: 14.05 ± 0.88) were enrolled in the study. The mean duration of the disease between diagnosis and cardiac assessment was 8.7 ± 1.2 months (5–14 months).

Children with other risk factors such as obesity, diabetes, and renal or liver disease and those who were taking oral medications that may affect the cardiovascular system were excluded from the study. Patients for whom the echocardiographic image quality was poor were also excluded. 

### 2.2. Evaluation of the severity of psoriasis

The severity of psoriasis was assessed using Psoriasis Area and Severity Index (PASI) scores, which have a range between 0 and 72, scores over 10 indicating moderate-severe disease (5).

### 2.3. Echocardiographic measurements

All patients underwent an echocardiographic examination using the Philips IE33-model echocardiography machine (Phillips Medical Systems, Andover, MD, USA), equipped with a 5 MHz sector and 7–11 linear transducers. Two-dimensional echocardiographic images were obtained using apical four-chamber (4C) and 2C views and a parasternal long axis (LAX) view. 

### 2.4. Left ventricular functions

Left ventricular dimensions, interventricular septum diastolic (IVSD) and left ventricular posterior wall diastolic (LVPWD) thicknesses, and left ventricle end-diastolic (LVED) and left ventricular end-systolic (LVEDS) dimensions were measured and left ventricular ejection and shortening fractions (LVEF-SF) were calculated by using the Teichholtz formula (6). Left ventricular diastolic functions were studied using pulse wave echocardiography using mitral inflow Doppler velocities; early (E wave) and late (A wave) velocities, deceleration time, and isovolumetric relaxation time (IVRT) were measured. The E/A ratio was calculated.

TDI was used for assessment of left ventricular myocardial functions. The velocities were obtained from the lateral mitral annulus throughout the cardiac cycle; early and late diastolic myocardial velocities (E’ and A’), systolic velocity S’, IVRT, and isovolumic contraction time (IVCT) were measured. The myocardial performance index (MPI; Tei index) was calculated by using the formula IVRT + IVCT/LVET.

Left ventricular global and longitudinal strain was studied by semiautomated speckle-tracking echocardiography. Images of the apical 4C, 2C, and long axis views were optimized at a frame rate of 60 frames/s. The systole was defined in the apical long axis view by opening and closure of the aortic valve, simultaneous with the peak of the T wave on electrocardiography (7). 

### 2.5. Elastic properties of the aorta

Aortic (Ao) diameters were measured from the LAX view on M-mode tracing at a level of 3 cm above the aortic valve. The systolic diameter (AoSD) was measured at the maximum anterior motion of the aorta and the diastolic diameter (AoDD) was measured at the peak of the QRS complex on the simultaneously recorded ECG. Systolic blood pressure (SBP) and diastolic blood pressure (DBP) were measured by sphygmomanometry just before echocardiographic examination (4). The following indexes of aortic function were calculated:

· Aortic stiffness (AS)% = 100 × (AoSD – AoDD)/AoDD

· Aortic distensibility (AD) = 2 × (AoSD – AoDD)/(AoDD × PP)

Carotid intima media thickness (CIMT) was measured with the L-11 MHz linear transducer. CIMT measurements were obtained when the patient was lying in supine position and the neck was rotated to the opposite side of the examiner. The average of three measurements was used for statistical analysis. The CIMT was accepted as the distance between the interface of the lumen and intima and the interface between the media and adventitia (8).

FMD of the brachial artery was calculated by using the L-11 MHz linear array transducer after 10 min of resting in the supine position from the brachial artery. A nonbranching, straight segment of the brachial artery above the antecubital fossa was identified and images were recorded. Baseline brachial artery diameter was measured offline at the tip of the R-wave of simultaneous ECG tracings. A pneumatic cuff on the upper arm was then inflated up to 50 mmHg above systolic blood pressure for 5 min and then released. After cuff deflation, brachial artery diameter was measured at every 30 s for 3 min in the end-diastolic phase. FMD was calculated as the percentage change in diameter from baseline value to the biggest value after cuff deflation (9). 

### 2.6. Statistical analysis

Statistical analysis was performed using SPSS 20 for Windows (IBM Corp., Armonk, NY, USA). The independent-samples t-test or Mann–Whitney U test was used to compare study variables. For categorical variables the chi-square test was used. Correlations were evaluated by univariate and multivariate linear regression analysis. P < 0.05 was accepted as an indication of statistical significance.

## 3. Results

Patients and control subjects were similar in terms of age and sex. PASI scores were 7.0 ± 1.84, indicating moderate disease. The clinical and demographic characteristics of the groups are shown in Table 1. 

**Table 1:** Demographic characteristics of the patients and control group.

	Psoriasis patients (n = 20)	Healthy controls (n = 20)	P
Age (years), mean ± SD	14.2 ± 0.89	14.05 ± 0.88	>0.05
Sex	12 girls, 8 boys	12 girls, 8 boys	>0.05
Weight (kg)	52.5 ± 5.03	53.8 ± 3.61	>0.05
Height (cm)	154.1 ± 4.96	152.2 ± 3.87	>0.05
TA systolic	89.5 ± 1.73	89 ± 2.79	>0.05
TA diastolic	45.95 ± 5.28	42.7 ± 2.88	>0.05
HR (beats/min)	79 ± 3.46	75.7 ± 16.6	>0.05
PASI score	7.0 ± 1.84		
Disease duration (months)	8.7 (5–14)		
Medication	Topical		

PASI: Psoriasis Area and Severity Index, SD: standard deviation, TA: arterial tension.

In comparison of M mode echocardiographic measurements, IVSD and LVPWD were found to be thicker and LVEDD was larger in patients with psoriasis compared to controls (P < 0.05). LVESD, ejection fraction, and shortening fraction did not differ between the groups. In assessment of left ventricular diastolic functions, the velocity of the E wave was decreased and the A wave was increased significantly (P < 0.05). The E/A ratio was decreased in patients, indicating more restrictive left ventricles. Comparison of echocardiographic values is shown in Table 2.

**Table 2:** M-mode, pulse wave Doppler, tissue Doppler echocardiographic, and strain characteristics of the patients and control group.

	Psoriasis patients(n = 20)	Healthy controls(n = 20)	P
IVSD (mm)	0.71 ± 0.065	0.68 ± 0.04	<0.05 (0.01)
LVPWD (mm)	0.75 ± 0.041	0.73 ± 0.038	<0.05 (0.01)
LVESD (mm)	33.8 ± 0.07	34.3 ± 0.08	>0.05
LVEDD (mm)	45.7 ± 0.22	45.4 ± 0.13	<0.05 (0.02)
SF (%)	32.3 ± 1.8	32.8 ± 1.98	>0.05
EF (%)	70 ± 2.5	69 ± 2.41	>005
Mitral E	0.68 ± 0.07	0.76 ± 0.056	<0.05 (0.003)
Mitral A	0.43 ± 0.07	0.35 ± 0.06	<0.05 (0.003)
E/A	1.64 ± 0.22	2.17 ± 0.35	<0.05 (0.02)
DEC time	66 ± 8.9	63.3 ± 8.5	>0.05
TDI E’	0.62 ± 0.07	0.70 ± 0.02	<0.05 (0.01)
TDI A’	0.50 ± 0.031	0.39 ± 0.036	<0.05 (0.008)
E’/A’	1.34 ± 1.14	1.58 ± 1.88	<0.05 (0.02)
S	0.36 ± 0.033	0.36 ± 0.032	>0.05
IVRT	57.9 ± 13.3	55.9 ± 6.42	>0.05
IVCT	74.7 ± 6.65	75 ± 5.77	<0.05 (0.02)
ET	154.85 ± 3.57	137.95 ± 10.84	<0.05 (0.02)
MPI	1.00 ± 0.13	0.90 ± 0.13	<0.05 (0.04)
Circumferential strain	–21.3 ± 3.75	–23.6 ± 3.78	<0.05 (0.05)
Longitudinal strain	–19.8 ± 1.89	–23.4 ± 3.92	<0.05 (0.004)

E: Early diastolic mitral inflow velocity, A: late diastolic mitral inflow velocity, IVRT: isovolumetric relaxation time, IVCT: isovolumic contraction time, ET: ejection time, TDI: tissue Doppler imaging, IVSD: interventricular septum diameter, LVPWD: left ventricle posterior wall diameter, LVESD: left ventricle end-systolic diameter, LVEDD: left ventricle end-diastolic diameter, SF: shortening fraction, EF: ejection fraction, DEC time: deceleration time, MPI: myocardial performance index.

In the comparison of TDI parameters, the velocity of the E’ wave was decreased while the A’ wave was increased in the psoriasis group. Decreased E’/A’ values in the patient group also indicate an early result of restrictive left ventricle physiology (Table 2). The MPI was also increased in patients as an indication of impaired global ventricular function.

While there was a significant increase in terms of aortic stiffness (P < 0.05), no significant change was observed in terms of aortic distensibility. CIMT and FMD% values did not differ significantly between the groups (Table 3).

**Table 3:** Aortic elasticity properties, arterial functions, and two-dimensional global left ventricular strain parameters.

	Psoriasis patientsn = 20	Control groupn = 20	P
Aortic stiffness	0.32 ± 0.14	0.18 ± 0.089	<0.05 (0.002)
Aortic distensibility	5.36 ± 3.45	7.30 ± 4.74	>0.05
CIMT	0.49 ± 0.057	0.46 ± 0.056	>0.05
FMD 1’	2.88 ± 0.14	2.90 ± 0.30	>0.05
FMD 3’	2.93 ± 0.16	3.006 ± 0.23	>0.05
Circumferential strain	–21.3 ± 3.75	–23.6 ± 3.78	<0.05 (0.05)
Longitudinal strain	–19.8 ± 1.89	–23.4 ± 3.92	<0.05 (0.004)

CIMT: Carotid intima media thickness, FMD: flow-mediated dilation.

In assessment of left ventricular strain parameters of the groups, there was a significant difference between the groups in terms of both circumferential and longitudinal strain parameters (Table 2).

**Figure 1:**
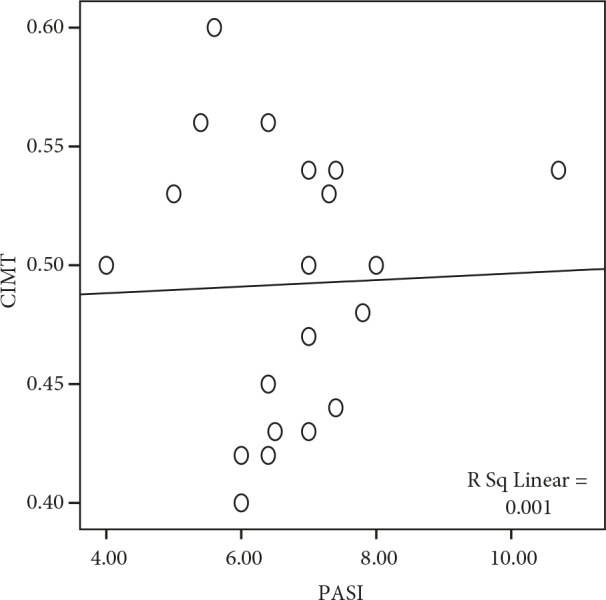
CIMT correlation with PASI. Positive correlation was found between CIMT and PASI score (r: 0.03).

**Figure 2:**
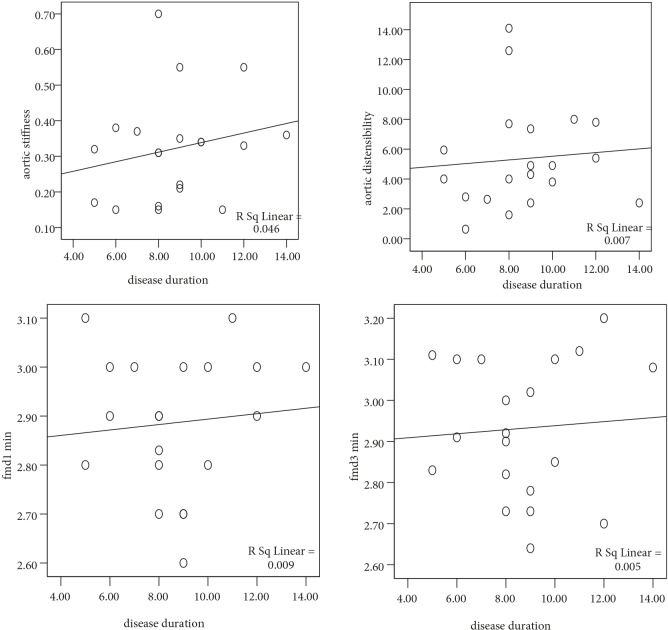
Correlation with disease duration. Disease duration was correlated positively with vascular parameters of aortic stiffness, aortic distensibility, FMD 1’, and FMD 3’ (r: 0.046, r: 0.007, r: 0.009, r: 0.005).

When we correlated clinical PASI scores with arterial functions, a positive correlation was found between CIMT and PASI scores (r: 0.03). Disease duration was correlated positively with vascular parameters of aortic stiffness, aortic distensibility, FMD 1’, and FMD 3’ (r: 0.046, r: 0.007, r: 0.009, r: 0.005) (Figures 1 and 2).

## 4. Discussion

Psoriasis is one of the most prevalent immune-mediated skin disorders. It is increasingly recognized to be related to cardiovascular events in adults (1). In the last decade several studies of adult patients with psoriasis have been carried out using echocardiography; however, no studies have been conducted in children previously (10). This study is the first study to describe the early effects of the disease in children.

The present study has shown that children with psoriasis had significantly hypertrophic and enlarged left ventricles compared to the healthy children. Saricaoglu et al. (11) reported increased left ventricular end-diastolic and end-systolic diameters in adult patients with psoriasis. Shang et al. evaluated patients by using conventional echocardiography and TDI and demonstrated subclinical LV dysfunction even in those without other cardiovascular risk factors (12). Our findings are in line with these studies and suggest that these ventricular changes start at an early age. 

Diastolic dysfunction may precede systolic dysfunction in the course of heart failure. We found impaired diastolic function by using pulse-wave Doppler and TDI. These findings may be early markers of diastolic dysfunction and restrictive physiology.

In this report we also evaluated aortic elastic properties. Factors known to affect aortic elasticity include age, sex, hypertension, diabetes mellitus, and coronary artery disease (13). None of our patients had any systemic disease other than psoriasis; therefore, the changes that we found in aortic elasticity and increased stiffness may be related to psoriasis and ongoing inflammatory processes. There was a significant positive correlation between aortic stiffness, distensibility, and disease duration. In adult studies, the degree of decrease in aortic distensibility, or increase in aortic stiffness, correlated with clinical severity and duration of the disease (4,14). We demonstrated these results even in childhood, indicating that immunologic processes are in charge even in the early stages.

The CIMT is another ultrasonographic parameter used for early detection of atherosclerosis risk. Shaharyar et al. (15) demonstrated increased CIMT in adult psoriatic patients. However, in our study the difference between study and control groups in terms of CIMT was not statistically significant. This may be related to relatively shorter duration of the disease that may cause detectable atherosclerotic changes in children. We also found a positive correlation between PASI scores and CIMT in our study group. This finding also supports our speculation about the shorter duration of the disease preventing the difference from reaching significant levels.

The vascular endothelium is an immunologically active organ. It regulates vascular tonus, secretes adhesion molecules, and plays a role in inflammation and angiogenesis. In the presence of hypertension, diabetes mellitus, obesity, oxidative stress, or dyslipidemia, endothelial dysfunction develops (16). FMD may be an early sign of endothelial dysfunction. We know that psoriasis sufferers with severe disease, as well as those with joint involvement, are more likely to have a greater systemic inflammatory burden with increased effects on the vascular system. In our study, albeit not reaching significance, decreased levels of FMD were observed even in the absence of these additional clinical involvements. We found a positive correlation between FMD 1’, FMD 3’, and disease duration. Balci et al. reported impaired endothelial function in adult psoriatic patients (17). Gonzalez et al. demonstrated that patients with arthritis without cardiovascular risk factors also exhibited endothelial dysfunction (18). In this study we demonstrated similar results that could be seen in childhood. 

Compared to conventional echocardiography, strain echocardiography provides a better measure of cardiac physiology and subclinical changes by evaluating global and longitudinal strain parameters (19,20). Wang et al. found that in early stages of heart failure, longitudinal strain is reduced while circumferential strain is preserved, because the subendocardial longitudinal fibers are primarily affected in psoriatic patients. With disease progression interstitial fibrosis develops, and circumferential strain impairment occurs later than longitudinal strain (21). We found that even with normal ejection fraction both longitudinal and circumferential strain were affected, indicating early subclinical dysfunction in psoriatic children. 

There are several limitations of this study. First, our sample size is small; we could not correlate these echocardiographic parameters with hematologic parameters, such as high-sensitivity CRP and lipid profile. Second, the disease duration is short, making it impossible to understand the long-term cardiac consequences of the disease. 

This is the first report of a study attempting to evaluate the early cardiac and vascular effects of psoriasis in children. Early identification of patients at risk and elimination of other contributing risk factors for prevention of long-term cardiac disease will prolong the life expectancy and improve quality of life in this population. Further studies are required to monitor cardiac and vascular health in childhood psoriasis.
